# Study of the Reaction Mechanism of the Excessive Adsorption of Mn^2+^ from Water by In Situ Synthesis of MnO_2_@SiO_2_ Colloid as an Adsorbent

**DOI:** 10.3390/ijms26072928

**Published:** 2025-03-24

**Authors:** Kun Wang, Yuchao Tang, Xinyu Zhang, Xianhuai Huang, Beiping Zhang

**Affiliations:** Anhui Provincial Key Laboratory of Environmental Pollution Control and Resource Reuse, Anhui Jianzhu University, Hefei 230601, China; wangkun@ahjzu.edu.cn (K.W.); 17730039472@163.com (X.Z.); huangxh@ahjzu.edu.cn (X.H.); beiping0107@163.com (B.Z.)

**Keywords:** in situ generation of MnO_2_@SiO_2_ colloids, adsorption for manganese removal, influencing factors, adsorption mechanism

## Abstract

An in situ-generated MnO_2_@SiO_2_ colloidal (ISMC) composite was used for the adsorption of Mn^2+^ ions in water. The adsorption capacity of ISMC at a concentration of 1 mg/L at 25 °C was as high as 3017.97 mg/g for the original concentration of 50 mg/L Mn^2+^ ions. Material characterization revealed that it is a porous sponge with a fibrous structure with a rough surface, many folds, and abundant pores, and these features provide many adsorption sites, which are conducive to the attachment of Mn^2+^ ions on its surface. ISMC has an isoelectric point of 3.5, indicating a negative surface charge that favors electrostatic attraction of Mn^2^⁺ ions. The surface hydroxyl groups provide additional active sites that allow for strong complexation with Mn^2^⁺ ions. Adsorption conformed to the Freundlich isotherm model (R^2^ > 0.98), suggesting multilayer adsorption, followed by pseudo-second-order kinetics (R^2^ > 0.98), with an optimum adsorption time of approximately 12 h. Low temperatures favor physical adsorption, whereas higher temperatures promote chemisorption via hydroxyl group complexation. The adsorption capacity increased with pH, which was attributed to the increased presence of surface hydroxyl groups. These findings highlight the significant potential of ISMCs for cation adsorption in water treatment applications.

## 1. Introduction

Mn^2+^ ions are prevalent contaminants in drinking water, often stemming from industrial emissions, agricultural practices, or dissolved substances in natural water sources [1,2,3]. Soluble manganese can accumulate on pipes, valves, and filters in water treatment systems, leading to blockage and reduced efficiency [4,5,6]. High concentrations of Mn^2+^ ions in drinking water can lead to unpleasant odors, degrade taste, and pose health risks to humans [7]. Long-term exposure to high concentrations of Mn^2+^ ions may even lead to irreversible Parkinson’s-like syndrome, known as manganese poisoning, which results in symptoms such as headache, nausea, vomiting, and neurological damage [8]. Consequently, the pollution of drinking water by divalent manganese ions poses a serious threat to water quality and human health [9]. The World Health Organization has set the maximum acceptable limit for manganese at 0.1 mg/L [10].

Common methods for removing manganese include oxidation [11], precipitation [12], filtration [13], ion exchange [14], and membrane separation [15]. Compared with other manganese removal methods, adsorption offers advantages such as high efficiency, wide applicability, no water quality restrictions, renewability, strong resistance to pollution, and simple operation. It is an environmentally friendly, economical, and feasible method for manganese removal that can be widely applied to various water sources to meet different needs [16,17,18]. Low-cost and high-efficiency adsorption materials have always been a focus of research. Studies on colloidal materials have shown that they possess nanoscale properties, good dispersibility, high surface energy, surface functionalization, and a porous structure [19,20]. These characteristics make them excellent adsorbents with broad application prospects in adsorption [21], catalysis [22], separation [23], and other areas.

Among the numerous adsorbents, MnO_2_ stands out for its excellent surface properties and has received widespread attention for its use in treating water containing heavy metals [24,25,26]. Research has shown that composite materials obtained through the chemical modification and complex design of MnO_2_ can significantly enhance the adsorption capacity [27]. MnO_2_@SiO_2_ colloidal (ISMC) is a multifunctional nanocomposite material in which MnO_2_ acts as the core and is encapsulated by a SiO_2_ shell. This structure endows ISMCs with unique properties and potential applications. As the core, MnO_2_ has excellent oxidation performance and catalytic activity, making it valuable in fields such as catalytic oxidation and environmental pollution control [28]. The SiO_2_ shell provides good chemical stability, thermal stability, and surface activity, enhancing the stability and controllability of the composite material [29]. Additionally, the SiO_2_ shell improves the dispersibility and solubility of the composite material, which is beneficial for its application in liquid phases [30].

This study synthesizes SiO_2_ powder by reacting (3-aminopropyl)triethoxysilane (APTES) with tetraethyl orthosilicate (TEOS). Mn^2+^ ions react with a KMnO_4_ solution to produce in situ-generated nano-MnO_2_ colloids. The two substances are sonicated to form ISMC particles, which are then used to adsorb Mn^2+^ ions in water. The adsorption capacities of in situ-generated MnO_2_ colloids, SiO_2_, and ISMCs for Mn^2+^ ions were compared. This study analyzes and discusses the effects of the mass ratio of MnO_2_ to SiO_2_ during colloid formation, the concentration of the ISMC, the adsorption temperature, the adsorption time, and the pH on the adsorption capacity of Mn^2+^ ions by the ISMC. Changes in the surface structure and functional groups of ISMCs before and after the adsorption of Mn^2+^ ions under different conditions were examined via scanning electron microscopy (SEM), X-ray photoelectron spectroscopy (XPS), Energy Dispersive Spectrometer (EDS), X-ray diffraction (XRD), Zeta potential, and Fourier transform infrared (FTIR). This research provides a reference for the adsorption mechanism of Mn^2+^ ions by ISMCs.

## 2. Results and Discussion

### 2.1. Sample Characterization

#### 2.1.1. SEM and EDS

**Figure 1:**
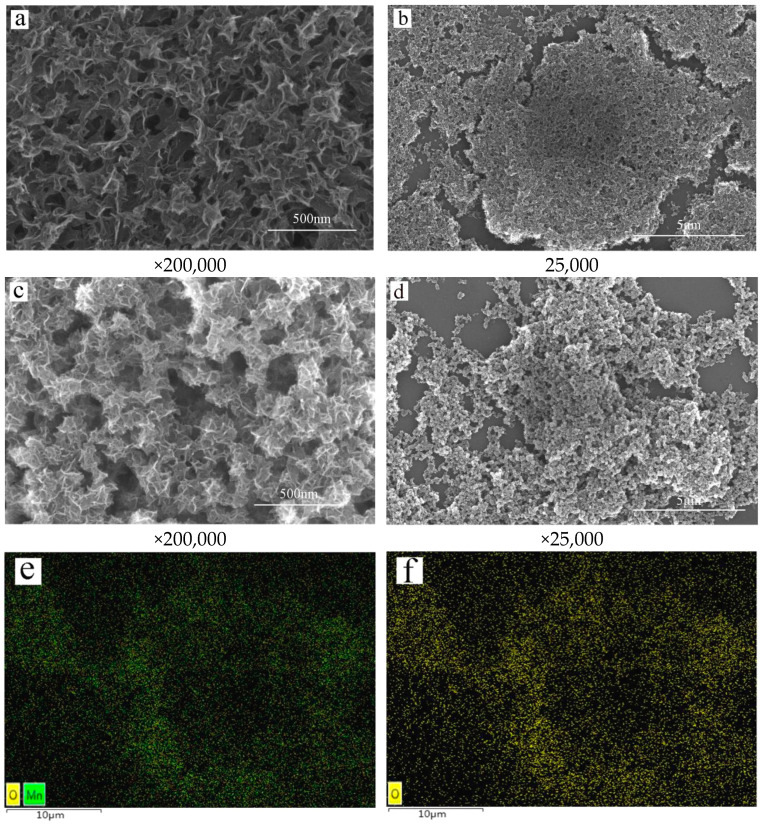

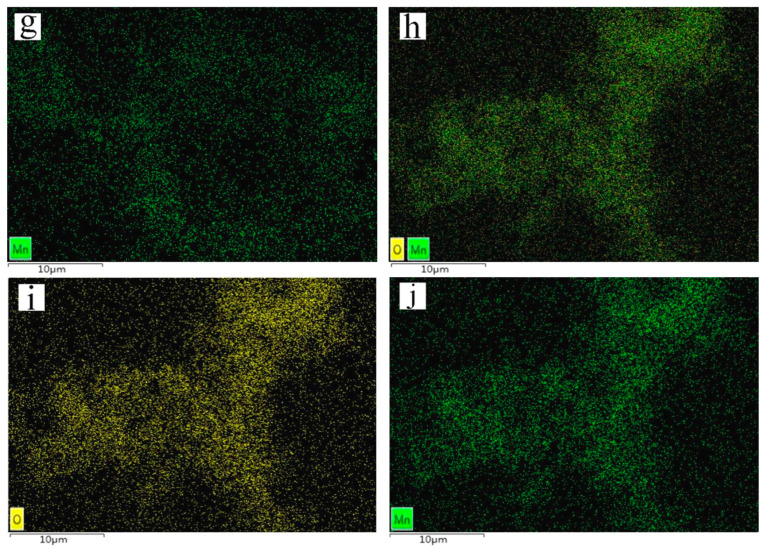
Scanning Electron Microscope images of the ISMC (**a**,**b**) before and (**c**,**d**) after adsorption; (**e**) total Mn, O elemental distribution before adsorption; (**f**) O elemental distribution before adsorption; (**g**) Mn elemental distribution before adsorption; (**h**) total Mn, O elemental distribution after adsorption; (**i**) O elemental distribution after adsorption; (**j**) Mn elemental distribution after adsorption.

The morphology of the ISMC before and after the adsorption of Mn^2+^ ions is illustrated in Figure 1a,b, which present the overall morphology of the ISMC before Mn^2+^ ion adsorption. The illustration reveals that the ISMC is a porous sponge with a fibrous structure characterized by significant surface roughness, numerous folds, and abundant open pores. The pore distribution appears dense and irregular, with a branching-like structure indicating multilevel structural characteristics. These features offer numerous adsorption sites and facilitate substance adhesion on the surface. Figure 1c,d show the morphology of the ISMC after Mn^2+^ ion adsorption. It is evident that the ISMC shape postadsorption is quasi-spherical, accompanied by a decrease in surface roughness and mesopores. The analysis indicates that upon Mn^2+^ ion adsorption on the ISMC surface, the ions occupy pores and concave structures, forming an adsorption layer [31]. With an increase in adsorbates, the surface tension of the adsorbed layer may lead to surface convergence toward minimum energy. The quasi-spherical shape postadsorption is attributed to the spherical structure having the smallest surface area [32], minimizing the surface free energy under tension.

The distributions of manganese and oxygen on the surface of the ISMC before and after Mn^2+^ ion adsorption are depicted in Figure 1e–j. The distribution of Mn and O on the surface of the ISMC prior to Mn^2+^ ion adsorption is relatively dispersed. The adsorption of Mn^2+^ ions induces chemical reactions on the surface, potentially altering the chemical state and surface structure. Consequently, this affects the interaction between Mn and O. It is plausible that Mn^2+^ ion adsorption may spatially bring Mn and O closer together, enhancing their interaction and facilitating aggregation. Furthermore, the adsorption of Mn^2+^ ions onto ISMC surfaces results in interactions with surface hydroxyl groups, subsequently modifying the surface charge and energy states. This process further promotes Mn and O aggregation.

#### 2.1.2. XRD

**Figure 2:**
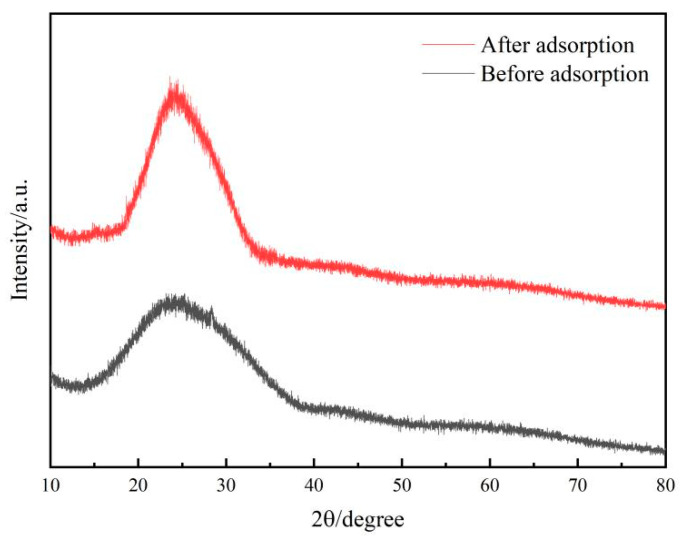
X-ray diffraction patterns of the ISMC before and after adsorption.

The X-ray diffraction (XRD) patterns of the ISMC before and after the adsorption of Mn^2+^ ions are shown in Figure 2. In the spectrum, peaks appearing at approximately 20–30° correspond to the (002) and (101) lattice planes, indicating the presence of Mn primarily in the form of δ-MnO_2_ within the ISMC [33]. The crystalline structure of the material is based on SiO_4_ tetrahedra, forming a three-dimensional network through vertex sharing, serving as a scaffold to guide MnO_2_ formation. The relatively weak diffraction peaks before adsorption compared to those after adsorption suggests that the material may be amorphous or be composed of nanoscale particles with a low degree of crystallinity [34]. Consequently, the ISMC possesses a large surface area, numerous exposed active adsorption sites, strong adsorption capacity, and high redox activity, rendering it capable of efficiently adsorbing Mn^2+^ ions [35]. The enhancement of the diffraction peak after adsorption indicates that the crystallinity of the material is enhanced after adsorption without significant displacement, which may be due to the adsorption of Mn^2+^ onto the surface of the material or into the pores through physical adsorption or surface complexation, resulting in the reorganization of the structure of the original material and the enhancement of the crystallinity, which is consistent with the SEM results.

#### 2.1.3. Zeta Potential

**Figure 3:**
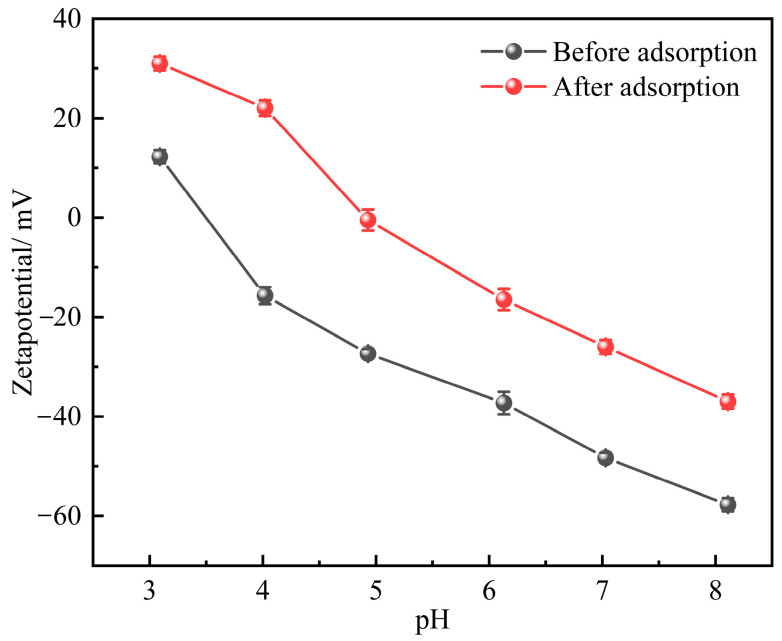
Zeta potentials of ISMCs in aqueous solutions at pH 3.09, pH 4.02, pH 4.93, pH 6.13, pH 7.03, and pH 8.11 before and after adsorption.

In this study, the pH and Zeta potential of ISMCs were measured before and after adsorption at pH = 3.09, pH = 4.02, pH = 4.93, pH = 6.13, pH = 7.03, and pH = 8.11. As shown in Figure 3, the isoelectric point of the ISMC before the adsorption of Mn^2+^ is 3.5. Under neutral conditions, the Zeta potential is −48.3 mV, indicating a negative surface charge. Compared with typical adsorbents, the ISMC has a lower Zeta potential [36,37]. This property enables the ISMC to adsorb Mn^2+^ ions through electrostatic interactions [38]. As the pH increased, the Zeta potential of the ISMC decreased, leading to a gradual increase in the negative charge, and the electrostatic adsorption of Mn^2+^ ions by the ISMC increased. This trend is consistent with the adsorption capacity observed at different pH values. After the adsorption of Mn^2+^ ions by the material, the pH decreased, and the isoelectric point increased to 5.0, which may be due to the release of protons and changes in surface charge properties, as well as potential complexation and hydrolysis effects. The above findings highlight the importance of electrostatic adsorption as a key mechanism for the adsorption of Mn^2+^ ions on ISMCs.

### 2.2. Conclusion of the Experiments

#### 2.2.1. Effect of the MnO_2_-to-SiO_2_ Mass Ratio in Pristine Materials and Comparison of the Adsorption Capacities of the Three Materials

**Figure 4:**
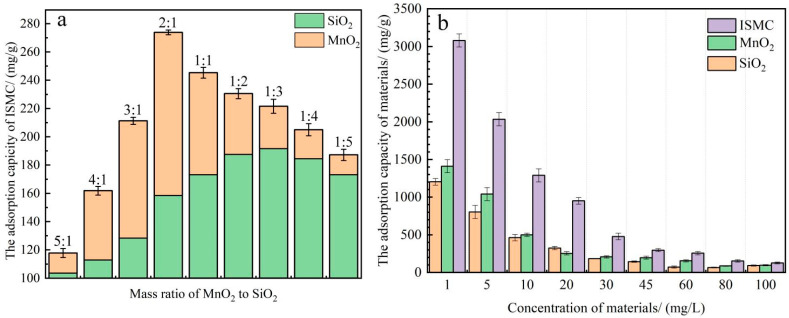
(**a**) Relationship between the adsorption capacity of Mn^2+^ and the mass ratio of MnO_2_ to SiO_2_ in the virgin material used to prepare ISMCs (reaction conditions other than those explored: [ISMC] = 45 mg/L, [Mn^2+^] = 50 mg/L, pH = 3.09, temperature = 25 °C, reaction time = 12 h). (**b**) Comparison of the Mn^2+^ ion adsorption capacities of three materials, SiO_2_, MnO_2_, and ISMC, under the same conditions (reaction conditions other than those explored: [Mn^2+^] = 50 mg/L, pH = 3.09, temp = 25 °C, reaction time = 12 h).

The adsorption capacity of the resulting ISMC for Mn^2+^ ions is shown in Figure 4a. The ISMC exhibited the highest adsorption capacity for Mn^2+^ ions when the mass ratio of MnO_2_ to SiO_2_ was 2:1. Because MnO_2_ is the main active component for Mn^2+^ adsorption and oxidation, if the content of MnO_2_ is too low, the active site is insufficient, limiting the adsorption capacity of Mn^2+^. SiO_2_, as a carrier, usually has a high specific surface area and pore structure, which can effectively disperse MnO_2_ and prevent it from agglomerating. If the content of MnO_2_ is too high, MnO_2_ is easy to agglomerate, and the specific surface area decreases, which reduces the diffusion of Mn^2+^ inside the material and leads to a decrease in adsorption capacity.

A comparison of the adsorption capacities of the three materials for manganese ions is presented in Figure 4b. The figure shows that the adsorption capacity follows this order: in situ-generated ISMC > in situ-generated MnO_2_ colloids > SiO_2_.

#### 2.2.2. Effect of Adsorbent Dosage, Concentration of Mn^2+^ Ions, and Temperature

**Figure 5:**
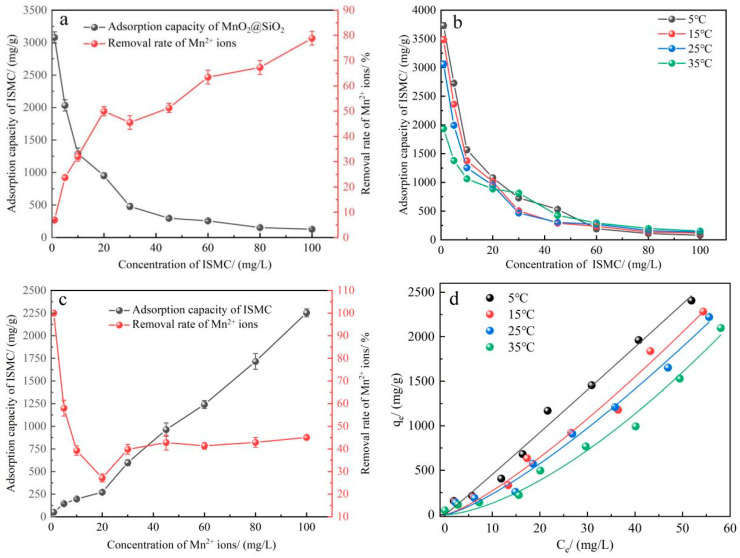
(**a**) Effect of the adsorbent dosage on the adsorption capacity (reaction conditions other than those explored: [Mn^2+^] = 50 mg/L, pH = 3.09, temperature = 25 °C, reaction time = 12 h). (**b**) In situ generation of ISMC adsorption capacity at different temperatures for different concentrations (reaction conditions other than those explored: [Mn^2+^] = 50 mg/L, pH = 3.09, reaction time = 12 h). (**c**) Effect of the Mn^2+^ concentration on the adsorption capacity (reaction conditions other than those explored: [ISMC] = 20 mg/L, pH = 3.09, temperature = 25 °C, reaction time = 12 h). (**d**) Freundlich model of ISMC for Mn^2+^ ions adsorption (reaction conditions other than those explored: [ISMC] = 20 mg/L, pH = 3.09, reaction time = 12 h).

Figure 5a shows the effect of the adsorbent concentration on the adsorption capacity. The figure shows that the adsorption capacity of ISMC decreases with increasing concentration. In a 1 mg/L ISMC solution, the adsorption capacity of colloidal particles reached 3017.97 mg/g. The analysis suggests that at high concentrations, ISMC particles may be more prone to aggregation, resulting in a reduction of the effective adsorption area available on their surface [39]. As the concentration of the ISMC increases, the active sites on its surface become occupied more rapidly. Concurrently, with increasing Mn^2+^ concentration, interactions between the active sites on the surface of the ISMC and the Mn^2+^ ions, such as van der Waals forces and electrostatic attraction, intensify. Consequently, the crowded environment around the active sites hampers the diffusion of Mn^2+^ ions to the surface of the ISMC. This competition for interactions ultimately leads to saturation effects [40].

Figure 5b shows that lower temperatures favor the adsorption of low-concentration ISMC, with a concentration of 1 mg/L ISMC resulting in an adsorption capacity of 3697.07 mg/g at 5 °C. This occurs for several reasons: low-concentration ISMCs possess a relatively large specific surface area and numerous adsorption sites, primarily relying on physical adsorption, which is typically an exothermic process [41]. The adsorption reaction releases heat, resulting in a higher adsorption capacity of low-concentration ISMCs at lower temperatures. Additionally, lower temperatures typically reduce thermal movement within the system and diminish competition between molecules of adsorbed substances [42]. On the contrary, high temperature favors ISMC adsorption at concentrations of 60 mg/L and above because high temperature promotes chemisorption, and the adsorption of Mn^2+^ ions by ISMC at high concentrations is mainly chemisorption.

Figure 5c shows the adsorption isotherm of the ISMC at 25 °C. The adsorption capacity of the ISMC for Mn^2+^ ions was found to increase with increasing Mn^2+^ concentration. When the concentration of Mn^2+^ ions is low, the active sites on the ISMC surface are not fully saturated. Conversely, as the concentration of Mn^2+^ ions increases, the active sites on the colloidal surface become partially saturated, and at this point, the interactions between the active sites on the colloidal surface become sufficiently strong [43]. As the concentration of Mn^2+^ ions continues to increase, the interactions between the already adsorbed Mn^2+^ ions induce further adsorption of Mn^2+^ ions onto the already adsorbed ions, forming a new adsorption layer [44]. Consequently, high concentrations of Mn^2+^ ions can result in multilayer adsorption. Furthermore, under conditions of elevated concentration, the concentration of Mn^2+^ ions is increased in the vicinity of the ISMC. This leads to an increased concentration gradient between the adsorbent surface and the surrounding fluid. Since diffusion is a process driven by the concentration gradient [45], the heightened concentration gradient enhances the adsorption capacity of the Mn^2+^ ions that diffuse to the ISMC surface.

The adsorption isotherms of the ISMC were subjected to Langmuir and Freundlich nonlinear fitting, with the results indicating a better fit with the Freundlich equation. The Freundlich model of ISMC for Mn^2+^ ion adsorption is shown in Figure 5d.

#### 2.2.3. Adsorption Isotherms and Adsorption Thermodynamics

The Freundlich fitting parameters are shown in Table 1. The adsorption of Mn^2+^ ions by the ISMC adheres to the Freundlich adsorption equation, suggesting the presence of diverse adsorption sites on the adsorbent, each with varying adsorption capacities. Moreover, the adsorption process is not singular but represents a multilevel adsorption phenomenon involving multiple mechanisms. Additionally, the adsorption process is reversible [46,47,48]. The adsorption process begins with the active sites on the surface of the ISMC attracting Mn^2+^ ions, marking the onset of the adsorption process. Owing to the negative charge of the active sites on the ISMC surface, they effectively attract positively charged Mn^2+^ ions, facilitating charge exchange with the surface of the ISMC. This leads to the formation of Mn-OH adsorbates on the ISMC surface, which coordinate with the hydroxyl groups on the active sites of the ISMC. Given the presence of multiple adsorption sites on the colloidal surface, the adsorption process manifests as multilayered, enabling ions to be adsorbed across different levels of the surface. With time, the adsorption process reaches an equilibrium state wherein the adsorption and desorption of ions achieve a dynamic balance. In the Freundlich adsorption equation, when n is less than 1, the adsorption process of ISMC is nonuniform and relatively complex, with variations among adsorption sites within the system [49]. Sites bearing hydroxyl groups exhibit higher adsorption capacities, whereas other sites reliant solely on physical adsorption demonstrate lower adsorption capacities.

The thermodynamic parameters analyzed in the Table 1 reveal that the entropy of the system decreases during adsorption, as evidenced by the negative value for ∆S. This negative entropy change indicates that the disorder within the system decreases during the adsorption process, implying that the adsorbed Mn^2+^ ions are ordered on the ISMC surface, i.e., in solution, the Mn^2+^ ions are freely dispersed and are in a highly disordered state, and they can move around and distribute throughout the solution system at random positions and directions, resulting in a higher entropy of the system. When Mn^2+^ is adsorbed onto the surface of ISMC, the ions are transformed from the free-dispersed state of the solution to the state of being immobilized at the adsorption site. A negative ∆H value indicates that the enthalpy of the system decreases during adsorption, indicating an exothermic adsorption process. Additionally, the negative Gibbs free energy change suggests that the adsorption process is spontaneous [50].

#### 2.2.4. Adsorption Kinetic Study

**Figure 6:**
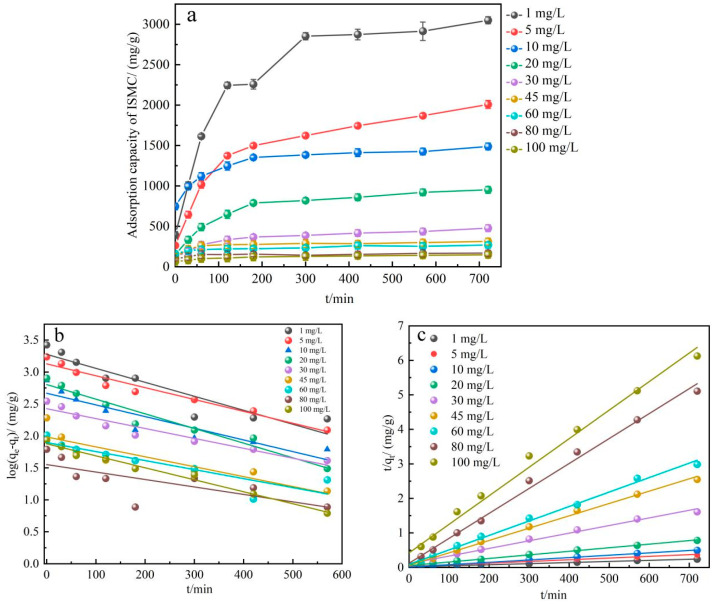
(**a**) Adsorption capacity of the ISMC for Mn^2+^ ions with different adsorption times. Fitted plots of (**b**) pseudo-first-order kinetics and (**c**) pseudo-second-order kinetics of Mn^2+^ ion adsorption by the ISMC. (Reaction conditions other than those explored: [Mn^2+^] = 50 mg/L, pH = 3.09, temperature = 25 °C).

As depicted in Figure 6a, the adsorption process seems to reach a near-completion stage after approximately 12 h, with the adsorption rate transitioning from rapid to gradual. It was determined that initially, the active sites of the adsorbent are relatively inactive, leading to a high adsorption rate. However, over time, as the concentration of Mn^2+^ ions in the solution decreases, the number of Mn^2+^ ions diffusing to the surface of the ISMC also decreases, consequently limiting the adsorption rate due to diffusion or mass transfer effects. Additionally, as adsorption proceeds, the pH of the solution decreases, causing an increase in H^+^ ions, which compete with Mn^2+^ ions for adsorption sites. This competition results in a gradual decrease in the adsorption rate.

The adsorption data of ISMC at 25 °C for various time intervals were fitted via pseudo-first-order and pseudo-second-order kinetic equations via linear regression analysis. The fitting results are depicted in Figure 6b for the pseudo-first-order model and in Figure 6c for the pseudo-second-order model. The adsorption process of Mn^2+^ ions by the ISMC aligns more closely with the pseudo-second-order kinetic equation, as depicted in the figure. Pseudo-second-order kinetic fitting parameters for the adsorption of Mn^2+^ ions by the ISMC is shown in Table 2. The pseudo-second-order kinetics model elucidates the correlation between the adsorption rate and the saturation level of adsorption sites throughout the adsorption process. This model is suitable for scenarios involving multiple adsorption sites [51] and can also capture the chemical interactions between adsorbed substances and ions at the adsorption sites. This observation suggests the presence of multiple adsorption sites on the ISMC, facilitating the adsorption of Mn^2+^ ions through diverse pathways. These sites not only adsorb Mn^2+^ ions but also engage in chemical reactions, forming Mn–OH bonds or other chemical complexes. Moreover, the pseudo-second-order adsorption kinetic equation accounts for the impact of adsorption site saturation. Initially, with ample adsorption sites available, Mn^2+^ ions are swiftly adsorbed onto the ISMC surface. However, as adsorption progresses, site saturation may occur gradually, leading to a deceleration in the adsorption rate. This phenomenon underscores that the adsorption rate is contingent not only on the concentration of unadsorbed substances but also on the saturation level of the adsorption sites [52].

#### 2.2.5. Effect of pH

**Figure 7:**
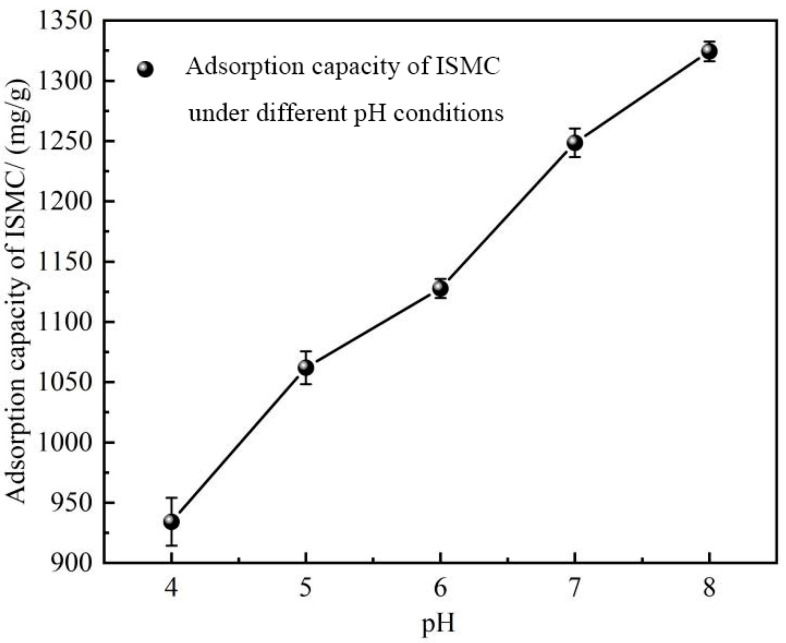
Effect of pH on the adsorption capacity of ISMCs. (Reaction conditions other than those explored: [ISMC] = 20 mg/L, [Mn^2+^] = 50 mg/L, temp = 25 °C, RT = 12 h).

Figure 7 shows the adsorption capacity under different pH conditions. These findings revealed a significant increase in the adsorption capacity of the ISMC upon pH adjustment. The pH of the solution before adsorption was 4.05, and after adsorption the pH of the solution decreased to 3.52. The alkaline conditions provided a substantial number of hydroxyl groups for the Mn^2+^ ions to form complexes with the hydroxyl groups on the surface of ISMC. This process continued to increase the adsorption capacity of ISMC until equilibrium was reached. Conversely, the high concentration of hydroxyl ions reacted with the oxygen (O) and manganese (Mn) atoms on the surface of the ISMC to form Mn-OH groups with a stronger affinity, which were able to adsorb Mn^2+^ ions more effectively. The aforementioned chemical reactions increase the number of surface active sites and improve the adsorption capacity. Under high pH conditions, the hydroxyl groups on the surface may undergo partial dissociation, leading to the formation of negative charges. This increases the negative charge density on the surface of the ISMC, thereby mitigating the charge repulsion effect and facilitating the adsorption of Mn^2+^ ions. The decrease in H^+^ concentration in solution reduces the competition for adsorption on the active sites, thereby enhancing the adsorption of Mn^2+^ ions by ISMCs under high pH conditions. Moreover, increased pH increases the chemical reactivity of Mn^2+^ ions in solution, potentially leading to morphological changes or the generation of complexes with OH^−^ ions in solution [53]. These alterations render the Mn^2+^ ions more prone to adsorption by the ISMC.

### 2.3. Adsorption Mechanism

#### 2.3.1. XPS

**Figure 8:**
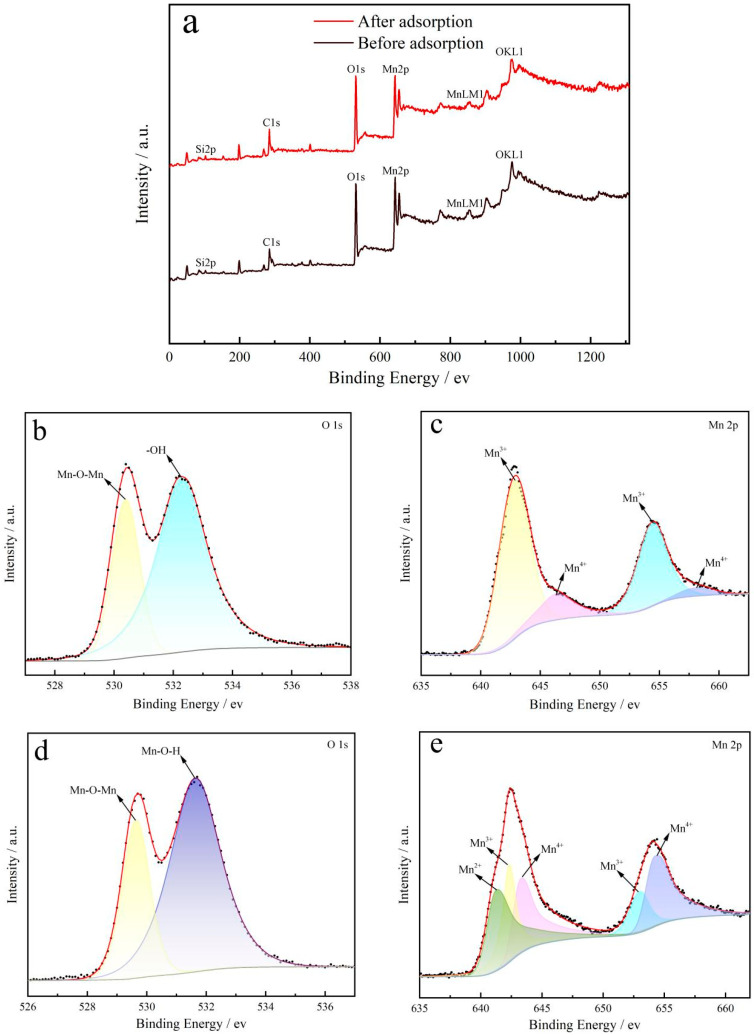
(**a**) Full X-ray photoelectron spectroscopy spectra of the ISMC before and after Mn^2+^ ion adsorption. (**b**) O 1 s X-ray photoelectron spectroscopy spectrum of the ISMC before the adsorption of Mn^2+^ ions. (**c**) Mn 2p X-ray photoelectron spectroscopy spectrum of ISMC before adsorption of Mn^2+^ ions; (**d**) O 1 s X-ray photoelectron spectroscopy spectrum of ISMC after adsorption of Mn^2+^ ions; (**e**) Mn 2p X-ray photoelectron spectroscopy spectrum of ISMC after adsorption of Mn^2+^ ions.

Figure 8 shows the comprehensive XPS spectra before and after Mn^2+^ ion adsorption by the ISMC. In the ISMC, the binding energies at 530 eV and 642/654 eV are attributed to O 1 s and Mn 2p, respectively. The high-resolution XPS spectrum of Mn 2p reveals six distinct peaks at binding energies of 641.25 eV (Mn^2+^), 642.5 eV (Mn^4+^), 643.5 eV (Mn^3+^), 653.14 eV (Mn^3+^), and 654.29 eV (Mn^4+^). In the high-resolution O 1 s XPS spectrum, the peaks at binding energies of 529.7 eV, 531.7 eV, and 532.3 eV correspond to Mn-O-Mn, Mn-O-H, and -OH, respectively [54]. The figure shows that the surface of the ISMC prior to Mn^2+^ ion adsorption is characterized by a high density of hydroxyl groups. Moreover, the material has a higher concentration of Mn^3+^ ions than after adsorption. It can be hypothesized that Mn^2+^ ion adsorption may induce reduction or rearrangement on the ISMC surface, leading to altered Mn^3+^ ion concentrations postadsorption. The presence of Mn^2+^ ions on the surface after adsorption, along with the observation of Mn-O-H, suggests that surface coordination plays a significant role in Mn^2+^ ion adsorption on the ISMC.

#### 2.3.2. FTIR

**Figure 9:**
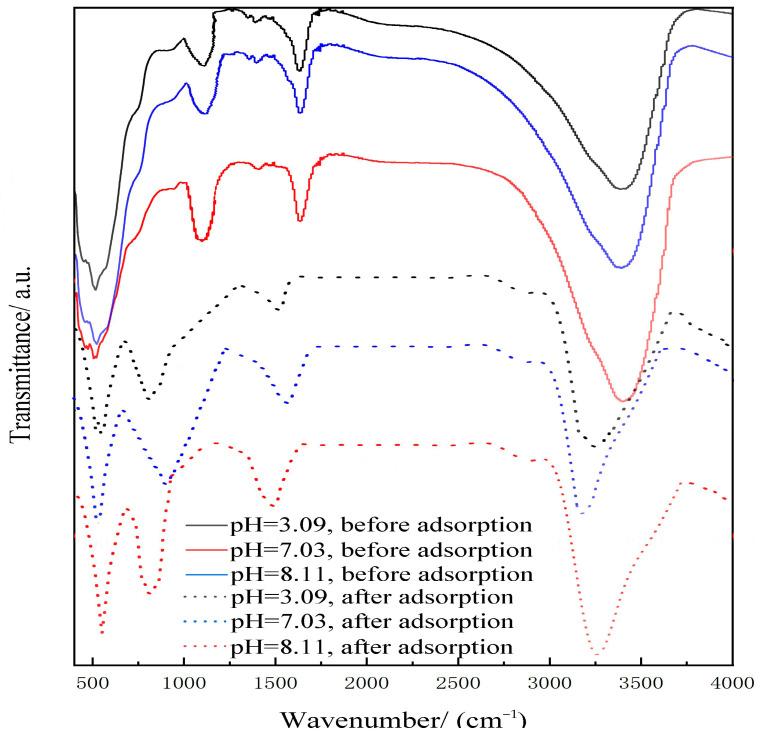
FTIR spectra of ISMCs before and after adsorbing Mn^2+^ ions at pH 3.09, 7.03, and 8.11.

Figure 9 shows the FTIR spectra of ISMC before and after adsorbing Mn^2+^ ions at pH 3.09, 7.03, and 8.11 at 25 °C. The surface of ISMC displays distinct vibrational peaks, including hydroxyl stretching vibrations at 3200–3300 cm^−1^, hydroxyl bending vibrations at 1000–1100 cm^−1^, the adsorbed water peak at about 1630 cm^−1^, the Mn-OH characteristic peak corresponding to 800–900 cm^−1^, and Mn-O lattice vibrations at 400–500 cm^−1^ [55]. Other peaks are attributed to impurity vibrations in the ISMC. The experimental results indicate that the hydroxyl vibrational peaks on the surface of ISMC gradually intensify with increasing solution pH. Concurrently, the relative content of metal–hydroxyl complexes formed also increases, leading to a higher Mn–OH content and a greater density of surface hydroxyl groups. This phenomenon is attributed to the alkaline conditions, which alter the charge nature of the ISMC surface, increasing its reactivity with OH^−^ ions in water and thereby facilitating the formation of hydroxyl groups [56]. The OH^−^ ions react with the manganese (Mn) and oxygen (O) atoms on the surface of the ISMC to form Mn-OH groups. The reaction process can be described as follows: MnO_2_ + 2OH^−^ → Mn-OH + O^2^⁻ + H_2_O. In this reaction, hydroxide ions combine with oxygen atoms on the surface of the ISMC, resulting in the formation of Mn-OH groups. These groups increase the hydroxyl group density on the surface of ISMCs and increase their ability to adsorb Mn^2+^ ions. This further indicates that surface coordination is a significant pathway for the adsorption of Mn^2+^ ions on ISMCs.

#### 2.3.3. Adsorption Process

**Figure 10:**
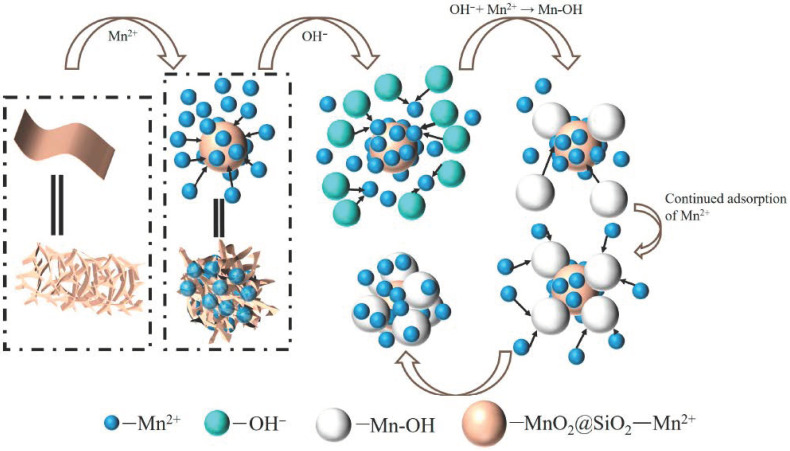
Adsorption reaction mechanism of ISMC for Mn^2+^ ions after increasing the pH.

Figure 10 shows the mechanism of Mn^2+^ ion adsorption by ISMC. The adsorption of Mn^2+^ ions by the ISMC composites involves a combination of electrostatic interactions, surface complexation, ion exchange, and aggregation deposition. The MnO_2_ component provides active sites for adsorption due to its redox properties and ability to interact with cations, whereas the SiO_2_ matrix offers a high surface area and structural support. The mesoporous structure of SiO_2_ allows efficient diffusion of Mn^2+^ to the embedded MnO_2_, further enhancing adsorption. After the solution pH increases, on the one hand, OH^−^ combines with Mn^2+^ in water to form Mn-OH, and the material continues to adsorb the Mn-OH formed in water. On the other hand, OH^−^ complex with Mn^2+^ already adsorbed on the surface of the material to form Mn-OH so that Mn^2+^ is more firmly adsorbed on the surface of the material. This results in the formation of a spheroidal composite material with a layer of Mn-OH wrapped around the outside, while the outer layer of Mn-OH can continue to adsorb the remaining Mn^2+^ in the water.

## 3. Experimental Section

### 3.1. Experimental Reagents

Manganese dichloride (MnCl_2_·4H_2_O, 99.0%), potassium permanganate (KMnO_4_, 99.5%), caustic soda (NaOH, 96.0%), (3-aminopropyl) triethoxysilane (C_9_H_23_NO_3_Si, 99.0%), tetraethyl orthosilicate (C_8_H_20_O_4_Si, 98.0%), potassium periodate (KIO_4_, 99.0%), potassium pyrophosphate (K_4_P_2_O_7_, AR), and sodium acetate (C_2_H_3_NaO_2_, 99.0%) were used. The above reagents were purchased from Sinopharm Chemical Reagent Co. in Beijng China and used directly without further purification. The reagents used in the experiments were prepared with ultrapure water (Sichuan Youpu Ultrapure Technology Co., Ltd. in Chengdu, China, 18.2 MΩ·cm).

### 3.2. Preparation of ISMCs

5 mL of APTES, 5 mL of TEOS, and 5 mL of water were added to the beaker. The mixture was stirred at 500 rpm for 30 min and then washed with ethanol. The mixture was centrifuged at 8000 rpm for 5 min, and the operation was repeated three times. Finally, the samples were dried and ground into powder to obtain the silane coupling agent-modified SiO_2_ powder.

**Figure 11:**
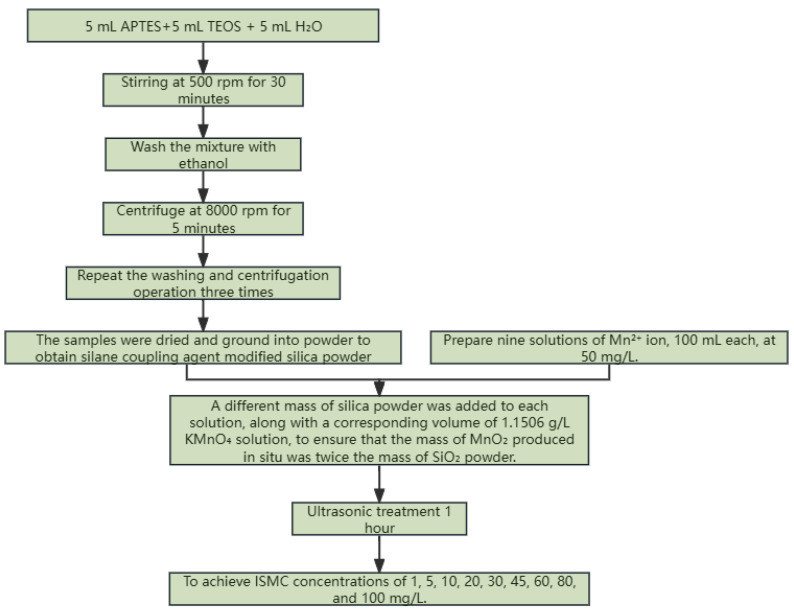
Flow chart of ISMC preparation.

A solution containing Mn^2+^ ions at a concentration of 50 mg/L and KMnO_4_ at a concentration of 1.1506 g/L was prepared. In the experiment, nine portions of Mn^2+^ ion solution, each with a volume of 100 mL and a concentration of 50 mg/L, were taken. Different masses of SiO_2_ powder were added to each portion. Subsequently, corresponding volumes of 1.1506 g/L KMnO_4_ solution were added to ensure that the mass of MnO_2_ generated in situ was twice the mass of the added SiO_2_ powder. Ultrasonication was then carried out for 1 h to achieve ISMC concentrations of 1, 5, 10, 20, 30, 45, 60, 80, and 100 mg/L. A flow chart for the preparation of ISMC is shown in Figure 11.

### 3.3. Characterization

The material solutions before and after the adsorption of Mn^2+^ ions were dried and gold sputtered, and then the morphology of the ISMC before and after the adsorption of Mn^2+^ ions was observed via a Hitachi SU8220 scanning electron microscope (SEM) and analyzed via EDS energy spectroscopy. They were then placed in a vacuum chamber and exposed to X-ray excitation, with a high-energy electron beam striking in the range of 10–30 keV. A Malvern ZS90 Nano Particle Size and Zeta Potential Analyzer (Dynamic Light Scattering (DLS)) was employed to conduct Zeta potential detection analysis. A laser diode (LD) was employed as the laser source, with a detection temperature of 25 °C and a refractive index of 1.845–1.955. The ISNC at different pH values for the detection of surface functional groups was analyzed via Fourier transform infrared (FTIR) spectroscopy with a Thermo Fisher-Nickelis10 instrument. The spectra were recorded with a silicon phenolphthalein (DTGS) detector as the light source and a mercury cadmium ammonium chloride (MCT) detector to detect the peaks at wavenumbers of 400–4000. A study was conducted to analyze the surface chemistry of ISMCs via X-ray photoelectron spectroscopy (XPS) on an ESCALAB250Xi instrument. The X-ray light source was an aluminum variety (Al Kα, 1486.6 eV), with an energy resolution of 20 eV and a photoelectron emission angle of 90°. To analyze the crystallinity of the absorbents before and after adsorption, X-ray diffraction (XRD) measurements were conducted using a Rigaku SmartLab 3 kW diffractometer. The X-ray source utilized was copper (Cu Kα). The scanning range was set from 10° to 80°, with a scanning speed of 5°/min. Notably, SEM, EDS, XPS, and XRD were not performed in the in situ generation state because of the limitations of existing detection techniques.

### 3.4. Determination Method of Mn^2+^ Ions

Mn^2+^ ions were determined via potassium periodate spectrophotometry. The UV–visible spectrophotometer used was a T6-1650E UV spectrophotometer manufactured by Beijing Spectrum Analytics General. After the reaction, the water sample was filtered through a 0.22 µm filter membrane and transferred to a 50 mL cuvette. It was then diluted to 25 mL with water. Next, 10 mL of the prepared potassium pyrophosphate–sodium acetate standard buffer solution was added and shaken well, followed by the addition of 3 mL of potassium periodate solution with a concentration of 20 g/L. The mixture was diluted with water, shaken well, and allowed to stand for 10 min. The absorbance was measured at 525 nm using a 5 cm cuvette with the reagents serving as a reference.

### 3.5. Batch Adsorption Experiments

#### 3.5.1. Calculation of the Adsorption Capacity

Mn^2+^ ions are not catalytically oxidized to MnO_2_ by dissolved oxygen in water in this scenario [57]. Consequently, the amount of ISMC adsorbed can be determined by subtracting the theoretically calculated oxidation removal amount from the actual Mn^2+^ ion removal amount in the system.

#### 3.5.2. Effect of the MnO_2_-to-SiO_2_ Mass Ratio in Raw Materials

Nine groups of 45 mg/L ISMCs were prepared by varying the mass ratio of MnO_2_ to SiO_2_ according to the method described in Section 2.2. These solutions were placed in a shaking chamber at a rotational speed of 200 rpm for 12 h at 25 °C, during which the remaining Mn^2+^ ions in the solution were adsorbed.

#### 3.5.3. Comparison of the Adsorption Capacities of the Three Materials

In the experiment, nine portions of 100 mL of a 50 mg/L Mn^2+^ ion mixture were prepared. Corresponding stoichiometric amounts of KMnO_4_ solution with a concentration of 1.1506 g/L were added to oxidize and adsorb the Mn^2+^ ions, resulting in in situ-generated MnO_2_ colloids at concentrations of 1, 5, 10, 20, 30, 45, 60, 80, and 100 mg/L. Additionally, nine portions of 100 mL Mn^2+^ ion solution with a concentration of 50 mg/L were prepared, and SiO_2_ powder at concentrations of 1, 5, 10, 20, 30, 45, 60, 80, and 100 mg/L, obtained via the method described in Section 2.2, was added. Nine groups of solutions were prepared according to the methods described in Section 2.2. These 27 samples were placed in a constant-temperature shaking chamber at rotational speeds of 200 rpm and 25 °C for adsorption over 12 h.

#### 3.5.4. Effect of Adsorbent Dosage

Nine groups of solutions were prepared according to the method described in Section 2.2 and allowed to adsorb for 12 h at 25 °C in a thermostatic shaking chamber at 200 rpm.

#### 3.5.5. Adsorption Isotherms and Thermodynamics

Nine conical flasks were prepared by adding 100 mL of Mn^2+^ solution at various concentrations. Subsequently, 0.67 mg of SiO_2_ powder prepared in Section 2.2 and 672.37 µL of 1.1506 g/L KMnO_4_ solution were added. The concentration of Mn^2+^ solution added should meet the requirement that the concentration of Mn^2+^ remaining in the solution after the reaction with KMnO_4_ was 1, 5, 10, 20, 30, 45, 60, 80, or 100 mg/L. After the nine conical flasks were sonicated for one hour, 20 mg/mL ISMC was obtained. The samples were incubated at 200 rpm for 12 h at 25 °C in a thermostatic incubator.

The Freundlich adsorption equation is applicable in the case of multilayer adsorption and has the following general form (1) [58]. The thermodynamic parameters can be derived from Equations (2) and (3) [59], as illustrated in Table 3.

(1) $$q_{e}=K\cdot C_{e}^{\frac{1}{n}}$$

(2) $$InK=-\frac{∆H}{R\cdot T}+\frac{∆S}{R}$$

(3) ∆G=∆H−T·∆S

In the aforementioned equation, q_e_ (mg/g) represents the quantity of adsorbate adsorbed on the adsorbent at equilibrium, Ce (mg/L) represents the concentration of the adsorbed substance in the solution at equilibrium, K denotes the Freundlich adsorption constant, which is used to describe the relationship between the adsorption capacity and the solution concentration, n denotes the nonuniformity parameter of the Freundlich adsorption equation, which describes the nonuniformity of the adsorption process, ΔH denotes the standard enthalpy change in the adsorption process, T (K) denotes the temperature, R denotes the gas constant (8.314 J/(mol/K)), and ΔG denotes the standard free energy change in the adsorption process.

#### 3.5.6. Effect of the Adsorption Temperature

In accordance with the methodology outlined in Section 2.2, 36 groups of solutions were prepared and placed in a thermostatic shaking box at 200 rpm for 12 h at temperatures of 5 °C, 15 °C, 25 °C and 35 °C.

#### 3.5.7. Adsorption Kinetics Study

A series of solutions with ISMC concentrations of 1, 5, 10, 20, 30, 45, 60, 80, and 100 mg/L were prepared following the procedure outlined in Section 2.2. These solutions were subjected to an adsorption process at 25 °C for 12 h. The samples were extracted at 0, 0.5, 1, 3, 5, 7, 9.5, and 12 h of reaction, and the concentration of Mn^2+^ ions was measured.

The investigation of kinetics aids in understanding the trajectory and underlying mechanisms of a reaction. Pseudo-first-order kinetic models (Equation (4)) and pseudo-second-order kinetic models (Equation (5)) are widely utilized to study the adsorption mechanism and determine the rate constants associated with the adsorption reaction [60,61].

(4) $$\log\left(q_{e}-q_{t}\right)=\log q_{e}-\frac{k_{1}}{2.303}t\;$$

(5) $$\frac{t}{q_{t}}=\frac{1}{k_{2}q_{e}^{2}}+\frac{t}{q_{e}}$$

where q_e_ (mg/L) and q_t_ (mg/L) represent the adsorption capacity of the adsorbent for Mn^2+^ ions at adsorption equilibrium and at a specific adsorption time, respectively. k_1_ (h^−1^) and k_2_ (g/(mg∙h)) are the adsorption rate constants for the pseudo-first-order kinetic model and pseudo-second-order kinetic model, respectively.

#### 3.5.8. Effect of pH

The procedure outlined in Section 2.2 was utilized to prepare six sets of ISMC solutions with a concentration of 20 mg/L. The pH of each mixture was adjusted to 4.05, 5.16, 6.19, 7.03, or 8.26. The samples were subsequently collected after they were subjected to agitation at 200 rpm at 25 °C for 12 h, after which the concentration of Mn^2+^ ions in the system was measured.

## 4. Conclusions

In conclusion, the ISMC composites produced through ultrasonication exhibited superior adsorption properties for Mn^2+^ ions compared with both the in situ-generated MnO_2_ colloids and the silane coupling agent-modified SiO_2_ powder. The ISMC exhibited a reticulated structure with relatively low crystallinity, featuring abundant wrinkles and open pores. Under neutral conditions, the surface Zeta potential was measured at −48.3 mV, which is indicative of the substantial presence of hydroxyl functional groups on the surface and an abundance of active adsorption sites. These attributes enable the effective adsorption and removal of Mn^2+^ ions. The adsorption behavior conforms to the Freundlich adsorption model and aligns with the pseudo-second-order kinetic model. Thermodynamic analysis revealed negative values for ΔS, ΔH, and ΔG, indicating an exothermic and spontaneous adsorption process, ultimately enhancing system organization. Furthermore, the adsorption capacity of ISMC at low concentrations surpassed that at high concentrations. Notably, pH has a significant effect on the adsorption capacity of ISMC. In 20 mg/L ISMC solution, the pH of the solution was 4.05 before adsorption, and after adsorption, the pH of the solution was reduced to 3.52. Increasing the pH of the solution promoted the complexation of Mn^2+^ ions with the hydroxyl groups on the surface of the ISMC, which improved the adsorption capacity of the material.

## Figures and Tables

**Table 1:** In situ generation of ISMC adsorption of Mn^2+^ fitting the parameters of the Freundlich equation.

Adsorption Temperature/°C	k	*n*	R^2^
5	42.9111	0.9752	0.9887
15	14.9024	0.7942	0.9829
25	11.8464	0.7713	0.9886
35	3.7118	0.6446	0.9852

**Table 2:** Pseudo-first-order and pseudo-second-order kinetic fitting parameters for the adsorption of Mn^2+^ ions by the ISMC.

Concentration of ISMC/(mg/L)	Pseudo-First-Order Dynamic Model			Pseudo-Second-Order Dynamic Model		
	K_1_	q_e_/(mg/L)	R^2^	K_2_	q_e_/(mg/L)	R^2^
1	0.0050	1891.39	0.8676	0.000007	3147.44	0.9920
5	0.0043	1342.61	0.9599	0.000009	2044.05	0.9892
10	0.0042	465.93	0.8564	0.000041	1458.57	0.9985
20	0.0053	637.81	0.9547	0.000020	961.54	0.9921
30	0.0036	266.91	0.9293	0.000045	452.49	0.9893
45	0.0036	96.42	0.8029	0.000192	280.11	0.9966
60	0.0033	78.74	0.7943	0.000195	238.66	0.9948
80	0.0027	35.62	0.5378	0.004342	138.31	0.9943
100	0.0043	75.23	0.9827	0.001619	120.92	0.9880

**Table 3:** Thermodynamic parameters of ISMC adsorption of Mn^2+^ ions.

Parameters	T (K)	∆G (kJ/mol)	∆S (kJ/(mol·K))	∆H (kJ/mol)
ISMC	278.15	−8.61	−0.16	−53.88
	288.15	−6.98		
	298.15	−5.35		
	308.15	−3.72		

## Data Availability

The data presented in this study are available from the authors upon request.
